# Co-expressed mitochondrial genomes: recently masculinized, recombinant mitochondrial genome is co-expressed with the female – transmitted mtDNA genome in a male *Mytilus trossulus* mussel from the Baltic Sea

**DOI:** 10.1186/1471-2156-15-28

**Published:** 2014-02-28

**Authors:** Tomasz J Sańko, Artur Burzyński

**Affiliations:** 1 Genetics and Marine Biotechnology Department, Institute of Oceanology of Polish Academy of Sciences, Powstańców Warszawy 55, Sopot 81-712, Poland

**Keywords:** Transcriptomics, EST, Masculinization, Paternally inherited mtDNA, DUI, Doubly uniparental inheritance, mtDNA inheritance

## Abstract

**Background:**

Few exceptions have been described from strict maternal inheritance of mitochondrial DNA in animals, including sea mussels (Mytilidae), clams (Donacidae, Veneridae and Solenidae) and freshwater mussels (Unionoidae) order. In these bivalves mitochondria and their DNA are transferred through two separate routes. The females inherit only the maternal mitochondrial DNA whereas the males inherit maternal as well as paternal mitochondrial DNA, which is usually present only in gonads and sperm. The mechanism controlling this phenomenon is unclear but leads to the existence of two separate mitochondrial DNA lineages in a single species. The lineages are usually well differentiated: up to 20-50% divergence in nucleotide sequence. Occasionally, a maternal mitochondrial DNA can invade the paternal transmission route, eventually replacing the diverged M-type and lowering the divergence. Such role reversal (masculinization) event has happened recently in the *Mytilus* population of the Baltic Sea which consists of *M. edulis × M. trossulus* hybrids, but the functional status of the resulting mitochondrial genome was unknown.

**Results:**

In this paper we sequenced transcripts from one specimen that was identified as male carrying both the female mitochondrial genome and a recently masculinized mitochondrial genome. Additionally, the analysis of the control region has showed that the recently masculinized, recombinant genome, not only has an M-type control region and all coding regions derived from the F-type, but also is transcriptionally active along side the maternally inherited F-type genome. In the comparative analysis, the two genomes exhibit different substitution patterns, typical for the M *vs*. F genome comparisons. The genetic distances and ratios of non-synonymous substitutions also suggest that one of the genomes is transitioning from the maternal to the paternal inheritance mode, consistent with its recent masculinization.

**Conclusion:**

We have shown, for the first time, that the recently masculinized mitochondrial genome is active and that it accumulates excess of non-synonymous substitutions across its coding sequence. This suggests, that, under certain cytonuclear incompatibility conditions, masculinization may serve to restore the endangered functionality of the paternally inherited genome. This is also another example of a mitochondrial genome in which the recombination in the control region predated its transition from paternal to maternal transmission route.

## Background

In the animal kingdom mitochondria are commonly inherited through the maternal line (SMI – Strict Maternal Inheritance) [1] and their inheritance is clonal. The number of mitochondria within a single spermatozoa is much lower than in an oocyte. In mammals, during fertilization, the sperm mitochondria usually enter the ovum but are are ubiquitinated and enzymatically degraded [2]. It has been shown, that sperm mitochondria apparently do not persist beyond 48 hours after fertilization in female embryos of *Mytilus* mussel [3,4] but it is unclear whether they are stochastically lost or actively eliminated thereafter [5]. The mitochondria inheritance system of these bivalves is complicated by the Doubly Uniparental Inheritance (DUI) phenomenon, originally described in Mytilidae [6,7] but present also in other, distantly related bivalves such as some clams (Veneridae, Donacidae and Solenidae) [8,9] and the members of Unionoida order (freshwater mussels) [10,11]. Under DUI, the females are homoplasmic and pass their mitochondrial genome to all their progeny, as in SMI. Males, however, also pass their mtDNA but only to their male progeny. Most work concerning the fate of paternal mtDNA was done in *Mytilus*. The paternal mtDNA, if present in female tissues, is silent [12], and exists in very low concentration [13]. However, in male zygotes sperm mitochondria aggregate in only one blastomere from which gonadal tissue is shaped during embryo development [3,14]. Consequently, both genomes are present and expressed in the male germ line and only the paternal genome is present in sperm. Both genomes may also be present in the male somatic tissues, but primarily the F genome is expressed there [15,16]. The M genome evolves faster than the F genome and accumulates more non-synonymous substitutions. It has been postulated that this may be explained by either relaxed or even positive selection [17-20]. The mechanism of DUI still remains unclear, although theoretical models have been developed explaining most of the observed DUI features [21,22].

Members of the *Mytilus edulis* species complex tend to hybridize in areas of sympatry. Such a hybridyzation zone has been described in the vicinity of the Baltic Sea. The species inhabiting the Baltic Sea was long considered to be *M. edulis*. However, allozyme data have changed the paradigm suggesting that the Baltic Sea population should be considered *M. trossulus*, hybridising with North Sea *M. edulis* in Danish Straits [23]. When more molecular markers were taken into account, it turned out that the whole Baltic population must be considered hybrid, with mixed nuclear background [24,25] and strong, unidirectional introgression of *M. edulis* mtDNA, leading to the complete replacement of the *M. trossulus* mtDNA [25,26]. Furthermore, the highly divergent (typically 20% in *M. edulis*) M genome is present at low frequencies only and is replaced by far less divergent (up to 4%) genomes of F origin [27-29]. These genomes have mosaic structures, with a part of the control region (CR) derived from the typical, highly divergent M genome and the coding sequences derived from the typical F genome [28,30]. This apparent role reversal of the F genome invading the paternal transmission route has been called masculinization [21,31] and was reported also in *M. galloprovincialis* from the Black Sea [32,33]. These cases are, in the phylogenetic sense, quite recent. In other DUI animals the divergence between the two lineages is much higher, although if the DUI phenomenon is an ancient trait, then the role-reversals must have occasionally happened because the last common ancestor of M and F lineages is usually much younger than DUI itself [11,22]. The recentness of this process in the Baltic *Mytilus* gave an opportunity to study it in more detail. It has been postulated that CR sequences of the M origin are somehow involved in the paternal inheritance, and hence the CR recombination would be prerequisite for masculinization [30,32]. The discovery that in American *M. trossulus* the typical F genome has mosaic CR, despite not being masculinized [19,34], has somewhat lessened the strength of the argument. It has also raised the question how the masculinized genome can be recognized, without experimentally following its transmission route.

In this paper, we report divergence analysis of a co-expressed F and recently masculinized genome from a single male *M. trossulus* from Baltic Sea (Gulf of Gdańsk), for the first time applying EST (Expressed Sequence Tags) analysis to that type of genomes.

## Methods

### Collection of samples

Mussels were collected from the Gulf of Gdańsk (Southern Baltic Sea) at the end of April 2007. For *Mytilus sp.* it is the reproduction season and the adult individuals are full of ripe gametes just before spawning. The sex of each specimen was determined by microscopic examination of both sides, to exclude hermaphrodite individuals [13]. Overall 30 ripe male individuals were selected. Gill and mantle tissue samples of each individual were stored at -70°C.

### DNA isolation and screening

The first step in identifying specimens bearing recombinant, presumably masculinized genomes among morphologically identified males, was to extract total DNA using the CTAB method [35]. The control region (CR) fragment was then amplified using selective PCR primers developed in our laboratory [28]. First amplification was performed with AB32-AB16 primers. They have been used to detect rearranged genomes throughout the European range of *Mytilus* and do not amplify from the regular – non recombinant genomes [33]. The expected proportion [28] of examined males (10 individuals) gave a positive signal in this PCR. Then the long PCR was performed with MF12S and MFCO2 primers flanking the CR [33], for the selected 10 individuals. The length of this PCR product is indicative of the type of amplified genome: for the typical M genome, the PCR product is about 4600 bp long, whereas for typical F genome it is almost 4900 bp long. In recombinant genomes, the PCR products are longer; the difference depends on the number of 950 bp long repeat units present [28] and hence the number of repeats can be roughly estimated simply by comparing the lengths of the PCR products (Additional file 1). One of the individuals was selected for further analysis at random. To determine the sequence of the CR, the PCR products of the second amplification were ligated into the pUC19 vector (*Sma*I digested) and transformed into chemocompetent *Escherichia coli* DH5α host cells. Recombinant plasmids were isolated using the Plasmid Mini kit from A&A Biotechnology and then sequenced by Macrogene Inc. in Korea (Sanger method) from both ends.

### Preparation of cDNA library

For further analysis, central part of the mantle tissue containing gonads from one male individual bearing the recombinant mitochondrial genome was chosen. Total RNA was purified with GenElute™ Mammalian Total RNA Miniprep Kit (#RTN70, Sigma-Aldrich) including DNaseI (#EN0521, Fermentas) “on column” digestion step. Tissue was digested in the lysis buffer with proteinase K (#P2308, Sigma-Aldrich), 2-mercaptoethanol (#M3148, Sigma-Aldrich) and incubated for 30 minutes at 55°C. RNA was eluted twice.

A cDNA library was created in cooperation with the Max Planck Institute in Berlin-Dahlem, Germany. The library was created using CloneMiner^TM^ cDNA Library Construction Kit from Invitrogen. The cloning into an *E. coli* Gateway System and subsequent clone sequencing (Sanger method) was performed semiautomatically at the Max Planck Institute. The bioinformatic analysis of obtained EST data was performed at the Institute of Oceanology, Polish Academy of Sciences, Sopot.

### Bioinformatic analysis

Primary sequence reads were filtered using pregap4 software from the Staden Package [36]. Low quality sequences (Phred quality value <20), cloning vectors, primers as well as the polyadenylation tails, were automatically masked. To separate the mitochondrial transcripts from the nuclear, all sequences were compared by the estwisedb software (wise2 package) [37] to HMM profiles, which were built for *Mytilus* sp. mitochondrial genes using HMMER [38]. The positively identified reads were clustered in gap4 [36], and the resulting mitochondrial transcripts (mtEST) were BLASTed [39,40] against a local database of reference mtDNAs (GenBank and own data, Table 1). This second filtering step allowed identification of two reference genomes for further comparative analyses: F-BMt [28] and RF-Mg [33], both with less than 5% divergence from the mtESTs.

**Table 1:** **Completely sequenced mitochondrial genomes of *Mytilus *** **sp. used in comparative analyses**

**Species**	**Accession**	**Type**	**Code**	**Reference**
*Mytilus edulis*	NC_006161	F	F-Me	[41]
Baltic *Mytilus trossulus*	DQ198231	F	F-BMt	[26]
Baltic *Mytilus trossulus*	DQ198225	M	M-BMt	[26]
*Mytilus galloprovincialis*	FJ890849	F	F-Mg	[42]
*Mytilus galloprovincialis*	EF434638	RF	RF-Mg	[33]
*Mytilus galloprovincialis*	AY363687	M	M1-Mg	[43]
*Mytilus galloprovincialis*	FJ890850	M	M2-Mg	[42]

RF: Recombinant F genome.

Estwisedb selected transcripts were mapped (assembled) onto the corresponding mtDNA reference genomes (Additional file 2A), in gap4. Manual screening of each assembly did not reveal any cloning or PCR anomalies (Additional file 2B). The mtEST consensus sequences were extracted for each gene separately in gap4 (Additional file 2C), trimmed to coding open reading frame (ORF) (Additional file 2D), and concatenated in the same order as the genes in mtDNA (Additional file 2E). There was no sequence polymorphism (differences between high quality reads) within any of the contigs. All individual consensus sequences were deposited in GenBank (Accession numbers: KF220383–KF220405). To broaden the scope of the comparative analysis, three other genomes were used: F-Me [41], F-Mg [42] and M-BMt [26] (Table 1). The F-Me and F-Mg were potential outgroups rooting the clades containing mtEST, whereas the M-BMt was an outgroup for all F – like genomes compared in this paper. From all genomes, coding sequences were extracted, trimmed to the extent represented by mtESTs and concatenated (Additional file 2). All seven concatamers were then aligned in MEGA5 [44]. The nucleotide sequences were converted into amino acid sequences using the invertebrate mitochondrial genetic code (translation according to Codon Usage table five, NCBI) to eliminate the risk of not-in-frame gap insertions (MEGA5 ClustalW protein alignment under the default settings, with PAM weight matrix). For further analysis nucleotide sequences arranged by the amino acid alignment with all the gaps, stop codons and missing data removed were used. Nucleotide and amino acid divergence was determined in pairwise comparisons between all concatamers. Pairwise distance (Tamura-Nei model) as well as the disparity index (I_D_) test were calculated in MEGA5 [44] under the default settings. For the I_D_ test, a statistical Monte Carlo test (10000 replicates) was used to estimate the *P*-value.

The Ka/Ks ratios were calculated using KaKs_Calculator2.0 [45]. Input set consisted of all possible pairs of the seven concatamers. The computation was performed with the GY model (modified Hasegawa-Kishino-Yano) [46] of substitutions and assuming invertebrate mitochondrial genetic code. The model was selected using HyPhy1.0 [47] with default parameters, 4 rate categories (for both tests: Hierarchical and AIC) and *P* < 0.05. Also, the sliding window data analysis was performed for paired sequences with HyPhy1.0 (GTR model and 3 × 3 window setting).

For calculation of maximum likelihood (ML) tree, all seven concatamers were used. The tree was calculated using MrBayes-3.1.2 [48]. The M-BMt sequence was set as an outgroup. The analysis consisted of 4 runs with 4 chains. For each run three chains were heated and one was a cold chain. Each run consisted of 25 mln generations and sampling frequency was set at 10000 generations. This procedure was sufficient to achieve effective sample size (ESS) of at least 1900. The substitution model was set as a General Time Reversible model with gamma-distributed rate variation across sites and a proportion of invariable sites (GTR + Γ + I model, nst = 6). The 50% majority-rule Bayesian inference tree was derived from obtained data with the burnin of 25%. Afterwards, the resulting tree was drawn in FigTree 1.4 [49]. The posterior probability values are included as an indication of the support for key nodes.

All research described in the manuscript has been performed in compliance with the ethical guidelines regarding the experiments on animals.

## Results

In 10 morphologically identified males, CR amplification with AB32 and AB16 primers gave homogenous products with the length of about 950 bp. An individual bearing the relatively short recombinant genome was selected for further analysis (MF12S-MFCO2 product length of approx. 6850 bp, indicative of the presence of two repeats). The sequencing of the clone library and *in silico* analysis confirmed the presence of two AB32-AB16 repeats in the CR. Sequence comparison confirmed that the genome belongs to the 11a/15 or *mf*2 haplogroup described previously [28,33], mainly from the Baltic and Mediterranean Sea: the sequenced parts of the CR were identical to some of the previously described haplotypes from this haplogroup (data not shown).

**Figure 1:**
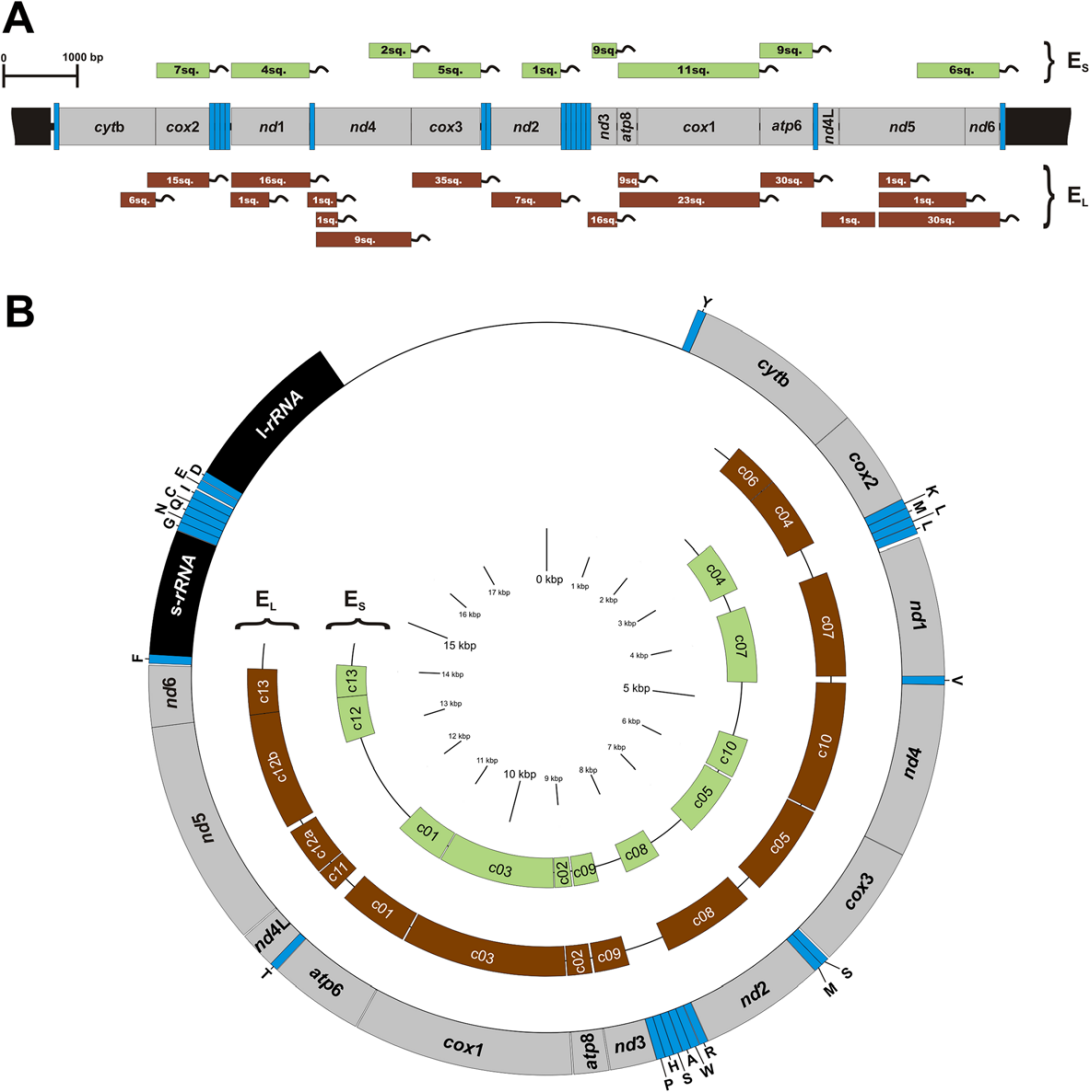
**Transcript mapping.** Figure **(A)** The two mtEST sets (E_L_ and E_S_) mapped on a hypothetical mitochondrial genome. Each green or brown rectangle represents a single contig. The numbers indicate the number of ESTs building each contig. Curved lines indicate the presence and positions of the polyadenine tails. Alternative polyadenylation sites and transcripts spanning two genes are retained. **(B)** The position of consensus mtESTs against a M*ytilus* circular mitochondrial genome. There are no sequence differences between apparently alternatively polyadenylated transcripts. Note that these mtESTs were further trimmed to the common coverage before performing comparative analyses.

The sequenced cDNA library from the selected specimen consisted of over 2300 ESTs and about 10.5% of them were identified as mtEST. They were clustered into 24 contigs. Each contig was assigned to one of the two sets of ESTs (presumably transcribed from two genomes), based on its distances from the two reference genomes (Table 2). The genome represented by 202 ESTs in 13 contigs closer to the RF-Mg genome was called E_L_ (large set of ESTs), whereas the second genome, containing 54 ESTs in 11 contigs was called E_S_ (small set of ESTs). Figure 1A is a schematic map of those two EST sets. Most genes were represented in both sets. The overall coding sequence coverage was 91.5% for E_L_ and 66.6% for E_S_ (Figure 1B). To avoid the bias associated with this difference, both sets and all reference sequences were trimmed to the longest common coverage and aligned. The alignment was 7515 bp (2505 amino acids) long and represented 64.1% of the mitochondrial coding sequence from 11 out of 13 mitochondrial protein coding genes (*cyt*b and *nd*4L were excluded because they were not present in the E_S_). There were no transcripts covering the recently described ORF in the control region [50] or the 16S rRNA subunit and there was only a single transcript for the 12S rRNA subunit (data not shown). Some genes with possible alternative polyadenylation sites and a few sequences spanning two adjacent genes have been identified but their presence did not influence the consensus generation.

**Table 2:** **Nucleotide *p*-distance (*d*** **±** ***S.E. × *** **10**^**–2**^**) between individual mtESTs from the two sets and the corresponding fragments of the reference genomes**

**EST set**	**Gene**	**F-BMt**	**RF-Mg**
**E**_**L**_	*cyt*b	1.48 (± 0.53)	0.63 (± 0.35)
	*cox*2	1.10 (± 0.38)	0.55 (± 0.26)
	*nd*1	1.78 (± 0.41)	0.47 (± 0.21)
	*nd4*	5.20 (± 0.60)	0.99 (± 0.27)
	*cox*3	3.31 (± 0.64)	1.60 (± 0.43)
	*nd2*	3.19 (± 0.56)	0.85 (± 0.30)
	*nd*3	2.85 (± 0.87)	0.85 (± 0.46)
	*atp*8	4.72 (± 1.27)	0.79 (± 0.53)
	*cox*1	2.60 (± 0.41)	0.78 (± 0.23)
	*atp*6	2.25 (± 0.52)	0.98 (± 0.35)
	*nd4*L	2.15 (± 0.92)	1.72 (± 0.83)
	*nd*5	2.61 (± 0.36)	1.03 (± 0.24)
	*nd*6	3.01 (± 0.76)	0.43 (± 0.30)
**E**_**S**_	*cox*2	0.00 (± 0.00)	1.53 (± 0.44)
	*nd*1	0.09 (± 0.09)	1.69 (± 0.37)
	*nd*4	0.18 (± 0.17)	6.91 (± 1.04)
	*cox*3	0.33 (± 0.19)	3.43 (± 0.61)
	*nd*2	0.00 (± 0.00)	3.28 (± 0.74)
	*nd*3	0.00 (± 0.00)	3.13 (± 0.92)
	*atp*8	0.00 (± 0.00)	4.05 (± 1.21)
	*cox*1	0.06 (± 0.06)	2.35 (± 0.39)
	*atp*6	0.00 (± 0.00)	2.39 (±0.55)
	*nd*5	0.15 (± 0.15)	2.01 (± 0.54)
	*nd*6	1.50 (± 0.54)	1.50 (± 0.54)

**Figure 2:**
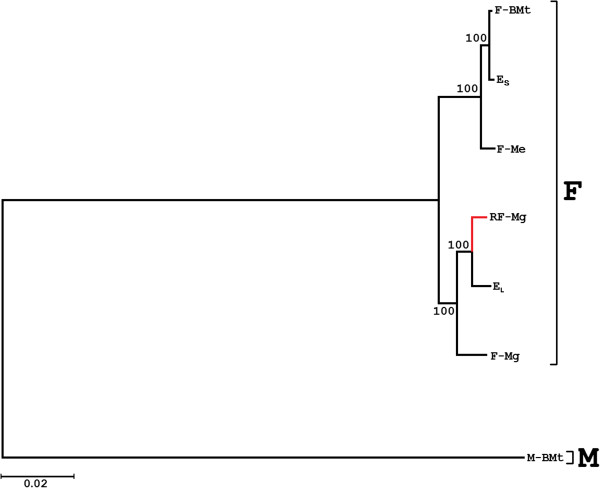
**Bayesian inference, majority-rule tree for mitochondrial transcripts of representatives from European *Mytilus *family.** Phylogenetic tree based on the 7515 bp long alignment of concatenated coding sequence fragments from the mitochondrial genomes expressed in the mantle of a male individual of Baltic *M. trossulus* with the reference genomes listed in Table 1. Branch support is given as posterior probability (Bayesian inference).

The phylogenetic tree based on the aligned set of representative gene fragments was inferred by the Bayesian approach (Figure 2). The relationships between all seven genomes were resolved with good support for all bipartitions. It confirmed the placement of both E_L_ and E_S_ genomes close to the other F-like genomes and the closest relationship of both genomes with the respective reference genomes (F-BMt and RF-Mg). This was confirmed also by the high resolution phylogeny involving more unpublished complete mitochondrial genomes (Additional file 3).

**Figure 3:**
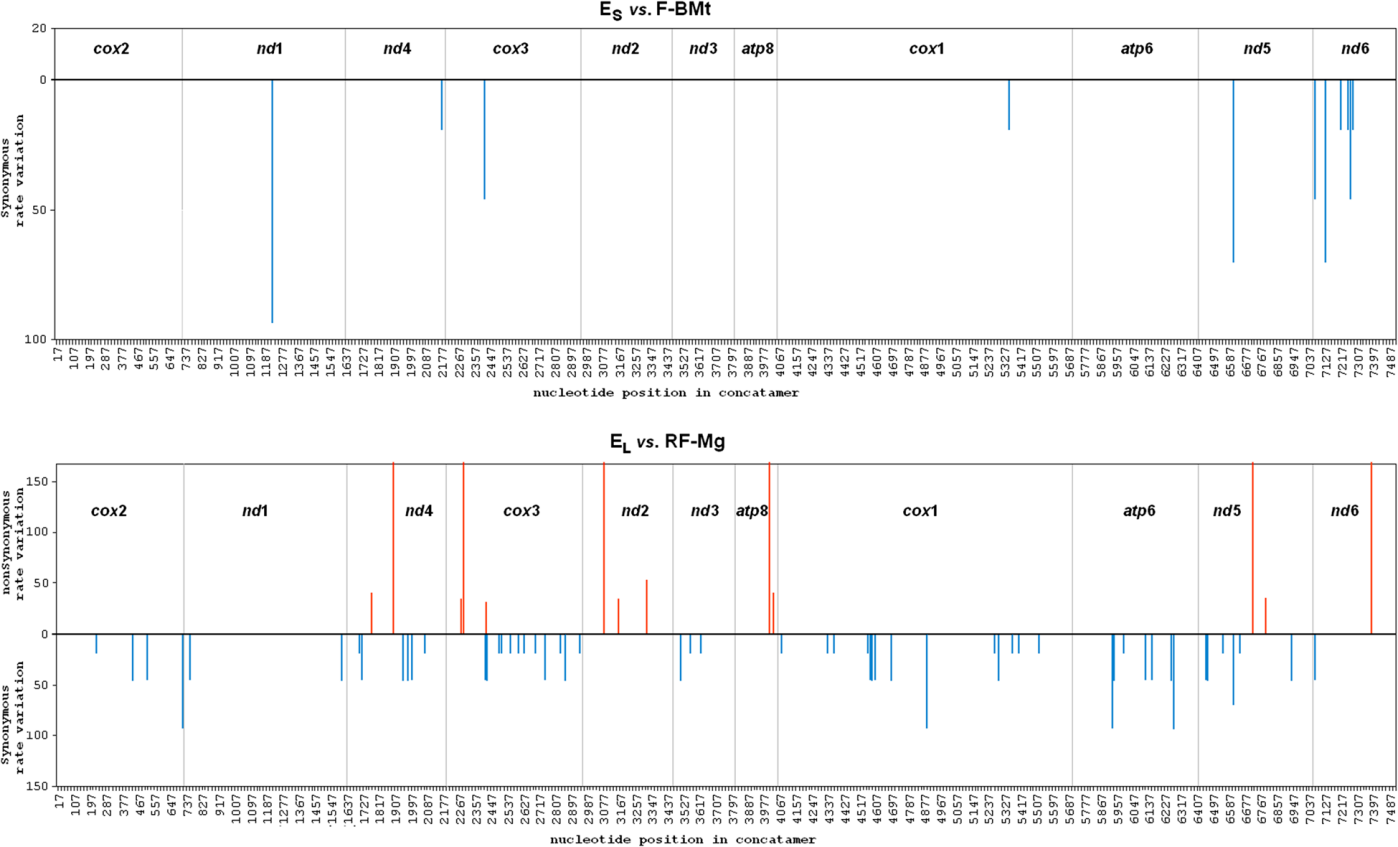
**Sliding window analysis of selective pressure.** Each codon was evaluated for its substitution patten. Negative values indicate purifying selection, positive values mark non-synonymous sites. Each of the expressed genomes was compared with its closest relative. Concatenated alignment was used in the analysis, gene boundaries are marked with thin vertical lines and clearly labeled.

Pairwise comparison of distances between all genomes (Table 3) showed an overall uniform pattern of synonymous and non-synonymous substitutions across all comparisons. This was in agreement with the disparity index (I_D_) test showing mostly homogenous substitution pattern, with only the outgroup M genome (M-BMt) exhibiting significant differences (Additional file 4). The only exception was the higher Ka/Ks ratio in E_L_:RF-Mg comparison (Table 3). It suggested elevation of the non-synonymous substitution rate along the recent history of the E_L_ genome. For similarly distant pairs of genomes involving the E_S_ genome (ea. the E_S_:F-Me comparison), the Ks values were similar but there were three times more non-synonymous substitutions in the E_L_:RF-Mg comparison. This seems to be representative for the paternally inherited genomes: if the two M genomes from *M. galloprovincialis* (M1-Mg and M2-Mg, Table 1) were compared in a similar way, the obtained Ka/Ks ratio was very similar (118.6 × 10^-3^). The sliding window, codon-by-codon analysis (Figure 3) showed that the sites responsible for this effect were spread along the whole alignment, intermixed with the numerous codons showing synonymous substitution bias.

**Table 3:** **Pairwise comparison of the concatenated mtEST sets and several published *Mytilus *** **mitochondrial genomes**

**Comparison**		**nt (aa)**	**Ka × 10**^**–3**^	**Ks × 10**^**–3**^	**Ka/Ks × 10**^**–3**^	***d *** **(± *S.E.*) × 10**^**–3**^
**E**_**S**_	F-BMt	12 (0)	0.00	5.33	0.00	1.60 (± 0.42)
	F-Mg	198 (19)	4.10	74.56	54.99	27.29 (± 1.93)
	RF-Mg	196 (15)	3.05	76.88	39.64	27.02 (± 2.15)
	F-Me	55 (4)	0.78	21.85	35.85	7.40 (± 0.95)
	M-BMt	1 669 (355)	92.32	1 859.44	49.65	278.14 (± 12.31)
	E_L_	202 (20)	4.10	76.41	53.64	27.89 (± 2.17)
**E**_**L**_	F-BMt	206 (20)	4.08	78.96	51.64	28.46 (± 1.06)
	F-Mg	125 (20)	4.01	44.03	91.11	16.99 (± 1.29)
	RF-Mg	65 (13)	2.59	21.51	120.25	8.77 (± 1.06)
	F-Me	218 (20)	4.02	87.35	46.04	30.19 (± 2.34)
	M-BMt	1 665 (361)	93.31	1 800.02	51.84	277.07 (± 11.86)
**F-BMt**	RF-Mg	200 (15)	3.06	78.39	38.98	27.59 (± 2.27)
	M-BMt	1 666 (355)	82.00	1 851.59	44.29	356.19 (± 20.19)

The number of nucleotide (nt) and amino acid (aa) differences, Ka and Ks values and ratios as well as the Tamura-Nei nucleotide distances (*d*) are shown. All Ka/Ks ratios are significantly lower than 1 (Fisher exact test p < 0.05).

## Discussion

We have shown co-expression of two moderately divergent mitochondrial genomes in the mantle tissue of a male *M. trossulus* from the Baltic Sea. Since the mantle consists of both generative and somatic tissues we expect one of the genomes to be the typical F genome, in line with the observed tissue-specific patterns of expression reported recently for the congeneric *M. galloprovincialis*[16]. Based on the comparative analysis we can conclude that the genome expressing the E_S_ set of transcripts must be the typical F genome of this individual. There were fewer E_S_ than E_L_ transcripts in the EST library suggesting that the sample may be enriched for the tissue preferentially expressing the second genome. Our main focus was on this second genome, represented by the E_L_ transcripts.

### Is the genome functional?

It is expressed, all mitochondrial protein coding gene transcripts are present and all the transcripts contain undisturbed ORFs. Moreover, they are typically polyadenylated near the ends of each coding sequence, although in the case of *nd*4L *- nd*5 *- nd*6 as well as *atp*8 *– cox*1 regions the presence of polycistronic transcripts cannot be excluded. Similar features have been reported for *M. galloprovincialis* mitochondrial transcripts recently [51]. Few sequences either did not contain the polyA sequence - simply because the sequencing did not reach it as only the ends of clones are sequenced - or contained it in unexpected places. There were two such cases in *nd*4 and one in *nd*5 sets. These can be viewed as cloning artifacts: they could have arisen either by mispriming from a particularly A-rich mitochondrial region during the first strand synthesis. They could have also be obtained as a result of attaching the polyA fragment to a partially degraded transcript at the ligation step during cloning. Long homopolymeric A or T DNA fragments are known to be particularly fragile [52], hence this interpretation is possible. Regardless of the origin, these sequences had no influence on the consensus calling – after removal of the terminal polyA they did not differ from the other transcripts mapped at the same mitochondrial region. Likewise the potential alternative polyadenylation sites observed may be artifacts of similar origin and without any consequences for the consensus. The lack of 16S sequences in our EST library may be moderately surprising, knowing that these transcripts are typically abundant in 454 transcriptome libraries [53], but the cloning procedure used during EST library preparation differs significantly from the one used in 454 sequencing. Apparently it is far easier for abundant sequences without polyA tail to ”leak-through” in 454 libraries [54]. We have found a single 12S transcript which apparently was polyadenylated. This is not surprising as polyadenylation of 12S transcripts has been reported for human mitochondria [55]. We can conclude that the E_L_ genome is most likely functional.

### Is the genome masculinized?

To conclude that a mitochondrial genome is masculinized it should ideally be detected in sperm and in the male offspring. This is rarely possible for various technical reasons. Usually the very presence of a genome in highly purified sperm can be viewed as an evidence that this is a male-transmitted genome [32]. The other approach is to follow its distribution among animals differing in gender [29]. Both approaches can be combined to some degree, allowing the use of poorer quality sperm [28]. This approach was initially received with reservations [56], the need to establish the true transmission route of these genomes was stressed. The E_L_ genome detected here clearly belongs to one of the haplogroups described by [28], based on the identity of sequenced CR fragments. On the other hand, the closest reference genome (RF-Mg) was isolated from a female *M. galloprovincialis* individual and therefore cannot be considered masculinized [33]. Further arguments supporting the masculinized status of the E_L_ genome include its very expression in male generative tissues. All experimental and model approaches to DUI agree that the paternally transmitted genome should be present and expressed there [3,4,13,16,22]. The second argument is associated with the observed pattern of substitutions: the E_L_ genome accumulated more non-synonymous substitutions than expected in the phylogenetic context. This is characteristic for a paternally inherited genome [17,21] and is not seen in the context of the second, E_S_ genome. Therefore the alternative hypotheses assuming that the E_L_ genome is not masculinized can be dismissed.

### What is the extent of recombination in the E_L_ genome?

The presence of mitochondrial genomes with mosaic CR sequences in the sperm of Baltic *M. trossulus*[28,30] strongly implied that these were masculinized genomes and that a reorganization of the CR involving the acquisition of sequences from the CR of the M genome was necessary for masculinization [22]. Since the CR of the E_L_ genome falls into this category it can be viewed as another case of a genome in which masculinization and recombination within the CR are linked. However, the confinement of the M-like sequences to the CR is important in the context of the original hypothesis. It remained possible that other parts of these molecules might have also been of type M [32]. This reservation is particularly true in the context of studies reporting recombination between different mitochondrial genomes outside the CR [57,58]. Here we show no ambiguity in the assignment of all transcripts to their genomes. In particular no M-like transcripts have been found. Therefore it is very unlikely that any other parts of the E_L_ genome are of M origin or that any products of potential recombination between E_L_ and E_S_ genomes are expressed. We can safely conclude that there was no physiologically important recombination outside the CR: no products of such recombination events were expressed at levels comparable with any of the parental sequences. The relationship between recombination and masculinization in the case of E_L_ genome can be further traced in the phylogenetic context. The closest reference genome (RM-Mg) does have a mosaic CR but there are no other reasons to consider this genome masculinized: it was localized in female tissues and its substitution pattern do not indicate the accelerated accumulation of non-synonymous mutations. Therefore we can parsimoniously assume that a single recombination event within the phylogenetic lineage leading to the E_L_ genome preceded its masculinization, the latter happening only very recently, possibly within a short period of the Baltic Sea existence. The mitochondrial dynamics of the Baltic Sea *Mytilus* population seems to be confined to this period [26].

### What is the driving force for the masculinization of mitochondrial genomes in Baltic population?

The most obvious explanation, that M genomes must occasionally be replaced because they degenerate, do not hold. The accumulation of potentially deleterious mutations in M-type genomes does not seem to be a problem for other DUI species. It has been shown that the system can be stable (ea. without any masculinization events) for hundreds of millions of years, as in unionidean mussels [10], moreover most *Mytilus* populations do not show masculinized genomes [33]. What is unique to the Baltic *M. trossulus* population is its nuclear background. Relatively high frequency of *M. edulis* alleles coupled with the complete replacement of the mitochondrial genomes creates a space for potential cytonuclear incompatibilities. They could lead to mitochondrial genome instabilities, both structural and functional. In fact, the CR length variants [59], recombination [30] and masculinization [27] can be viewed as manifestations of this instability. Under the mixed nuclear background the divergence threshold required to retain functionality of the mitochondrial genome may have abruptly lowered, rendering the divergent M genome less functional and therefore favoring masculinization. Studies reporting functional deficiencies of sperm carrying masculinized genomes in American *M. edulis*[60,61] seem to support such hypothesis. The reported fitness deficiency happened in the context of the presence of apparently native *M. trossulus* F genomes in *M. edulis* individuals. These genomes were originally interpreted as *M. edulis* masculinized genomes, but it has been shown that they are more likely typical F genomes of *M. trossulus*[19]. In this context it was most likely the cytonuclear incompatibility rather than masculinization that caused the fitness deficiency [19]. Whatever the reason, it clearly happened also in the context of interspecies hybridisation showing that under this conditions sperm mitochondria may become less fit.

### Can transcriptomics help to detect DUI?

There are three major F haplogroups in European populations of *Mytilus* spp. [18]. It has been noted previously, that a single lineage of genomes with mosaic CR structures has been derived from each of the haplogroups [28,33]. Two of the three recombinant lineages were associated with *M. galloprovincialis*. In the Baltic Sea, apparently very recent masculinization involved genomes from all three clades [28]. This is the reason why some of them are quite divergent – most of the divergence did not accumulate after the masculinization. The <4% divergence between the two expressed genomes observed in this study (E_L_ and E_S_) was high enough to unambiguously identify all transcripts. This shows the utility of transcriptomics in analysing mitochondrial divergence patterns. This methodology can be applied to other species as well, potentially overcoming the problems with the detection of DUI outlined by [9]: by analysing transcripts from male gonads one should be able to detect either single set of transcripts (for non-DUI species) or two sets of transcripts (indicating potential DUI species). We have shown that the sets can be distinguished even if the divergence is small. It should be even easier if the divergence is larger.

## Conclusions

We have shown that two mitochondrial genomes are co-expressed in the mantle of a male *Mytilus* mussel from the Baltic Sea. Both genomes are functional and one of them is recently masculinized. The masculinized genome contains a mosaic CR. The recombination was confined to the CR of its ancestor and preceded masculinization. These conclusions must be stated as tentative at present, given the sample size of one male. Nevertheless, the proposed methodology demonstrates the usefulness of transcriptome analysis in studying DUI.

## Abbreviations

Bp: Base pairs; CR: Control region; DUI: Doubly uniparental inheritance; F genome: Female type genome; mt: Mitochondria/mitochondrial; mtDNA: Mitochondrial DNA; mtEST: Mitochondrial Expressed Sequence Tag; M genome: Male type genome; ML: Maximum likelihood; ORF: Open reading frame; PCR: Polymerase chain reaction; poly(A): Polyadenylation; R genome: Recombinant F genome; SMI: Strict maternal inheritance; sq: Sequences.

## Competing interests

The authors declare no competing interests.

## Authors' contributions

TJS was the main researcher, performed specimen identification, DNA and RNA isolation, PCR, cloning, sequence assembly, and analysis, prepared the draft of the manuscript and all figures. AB designed the experiments, participated in the sequence analysis and assembly, edited the manuscript. Both authors read and approved the final manuscript.

## Supplementary Material

Additional file 1 **Primer binding map for Control Region identification.** A large fragment of the Control Region (CR) with flanking sequences was amplified with MF12S – MFCO2 primers spanning the region from *s-rRNA* to *cox2*. The presence of duplicated fragments was detected by amplification of the fragment between primers AB32 –AB16.These duplications contain M – derived fragments and are often present in masculinized genomes of European *Mytilus*. Blue, vertical lines indicate tRNA genes. Click here for file

Additional file 2: **EST consensus extraction scheme.** (A) All mtESTs were identified by BLAST (B) mapped on the reference genome (C) Stop codons, poli(A) tails and indels were removed and a consensus sequence was derived, (D) consensus as well as the reference sequence were trimmed to the same length; (E) All the consensus sequences were then concatenated according to the mitogenome order. Click here for file

Additional file 3: **High-resolution phylogeny analysis.** The tree was inferred based on nucleotide alignment (7515 bp long coding sequence) in MrBayes. The genome names as well as the E_L_ and E_S_ concatamers described in this paper had been marked on the branch tips. Red branches correspond to documented masculinized genomes. Abbreviations: *Me* – *Mytilus edulis*; *Mg* – *Mytilus galloprovincialis*; *Mt* – *Mytilus trossulus*; B*Mt* – Baltic *Mytilus trossulus*; M – male genome type; F – female genome type. Click here for file

Additional file 4: **The results of disparity index test (I**_**D**_**).** The test was performed in all pairwise comparisons in MEGA. Test values are above diagonal, statistical support (p values) are under the diagonal The *P*-values smaller than 0.05 (yellow marked) indicate significant rate heterogeneity. Click here for file
