# Supplementary figures and images for: Co-expressed mitochondrial genomes: recently masculinized, recombinant mitochondrial genome is co-expressed with the female – transmitted mtDNA genome in a male Mytilus trossulus mussel from the Baltic Sea
Source: BMC Genet. 2014 Feb 28;15:28. doi: 10.1186/1471-2156-15-28 (PMC3941564; doi:10.1186/1471-2156-15-28)

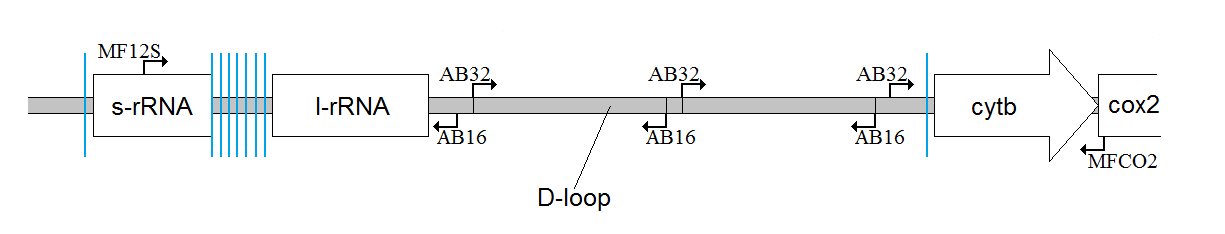

Supplement: Additional file 1 — Primer binding map for Control Region identification. A large fragment of the Control Region (CR) with flanking sequences was amplified with MF12S – MFCO2 primers spanning the region from s-rRNA to cox2. The presence of duplicated fragments was detected by amplification of the fragment between primers AB32 –AB16.These duplications contain M – derived fragments and are often present in masculinized genomes of European Mytilus. Blue, vertical lines indicate tRNA genes. [file 1471-2156-15-28-S1.png]

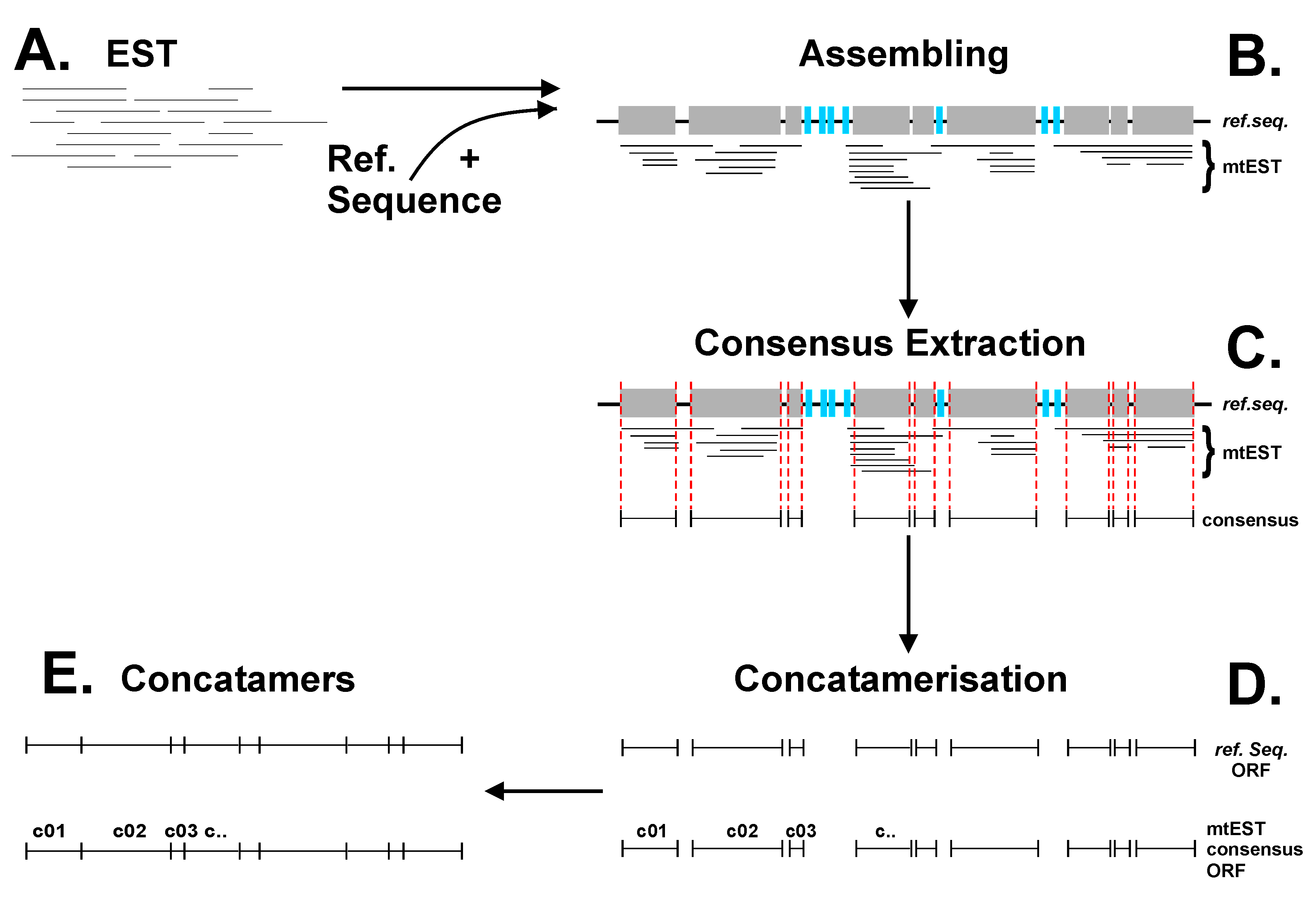

Supplement: Additional file 2: — EST consensus extraction scheme. (A) All mtESTs were identified by BLAST (B) mapped on the reference genome (C) Stop codons, poli(A) tails and indels were removed and a consensus sequence was derived, (D) consensus as well as the reference sequence were trimmed to the same length; (E) All the consensus sequences were then concatenated according to the mitogenome order. [file 1471-2156-15-28-S2.png]

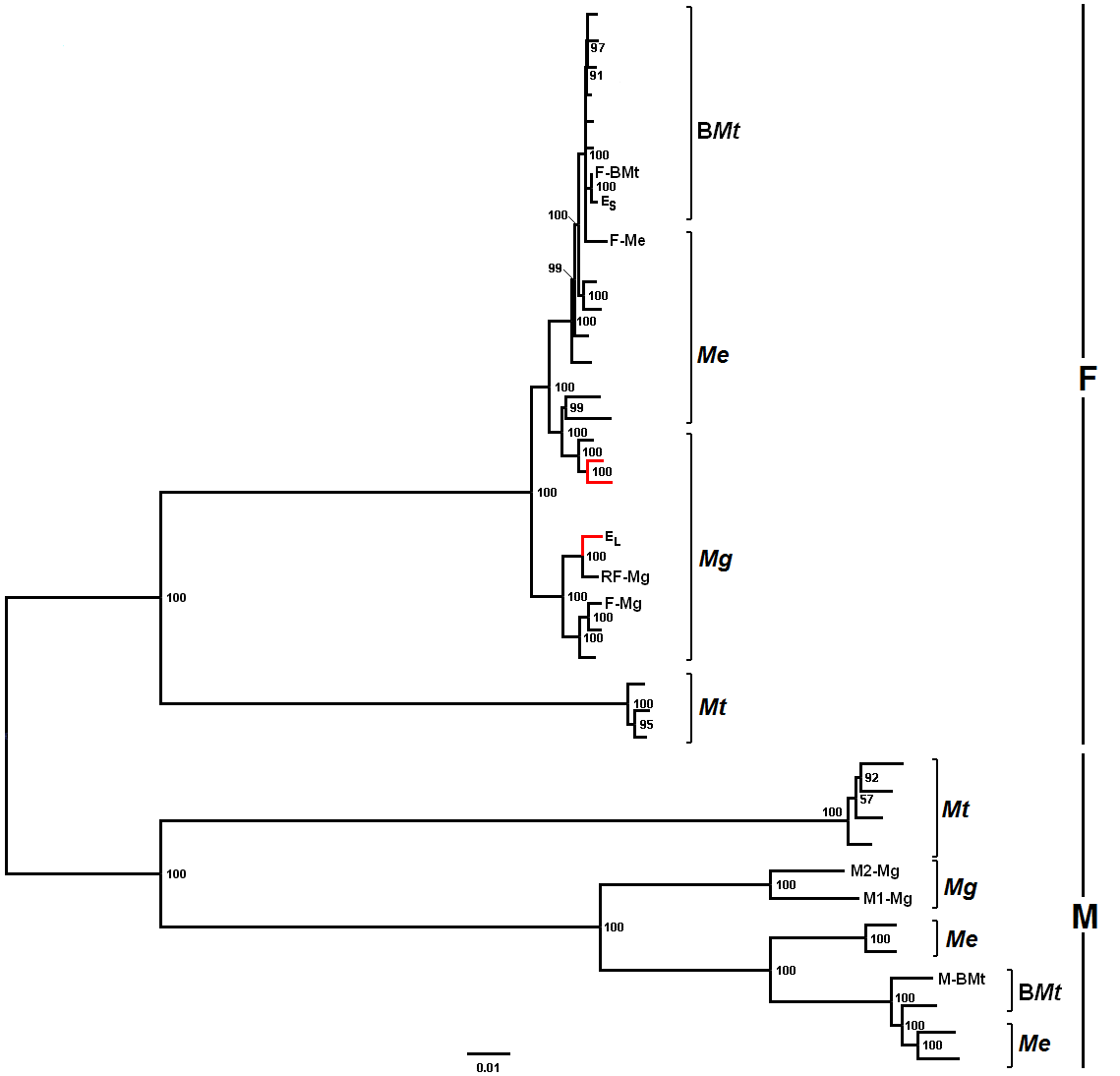

Supplement: Additional file 3: — High-resolution phylogeny analysis. The tree was inferred based on nucleotide alignment (7515 bp long coding sequence) in MrBayes. The genome names as well as the EL and ES concatamers described in this paper had been marked on the branch tips. Red branches correspond to documented masculinized genomes. Abbreviations: Me – Mytilus edulis; Mg – Mytilus galloprovincialis; Mt – Mytilus trossulus; BMt – Baltic Mytilus trossulus; M – male genome type; F – female genome type. [file 1471-2156-15-28-S3.png]

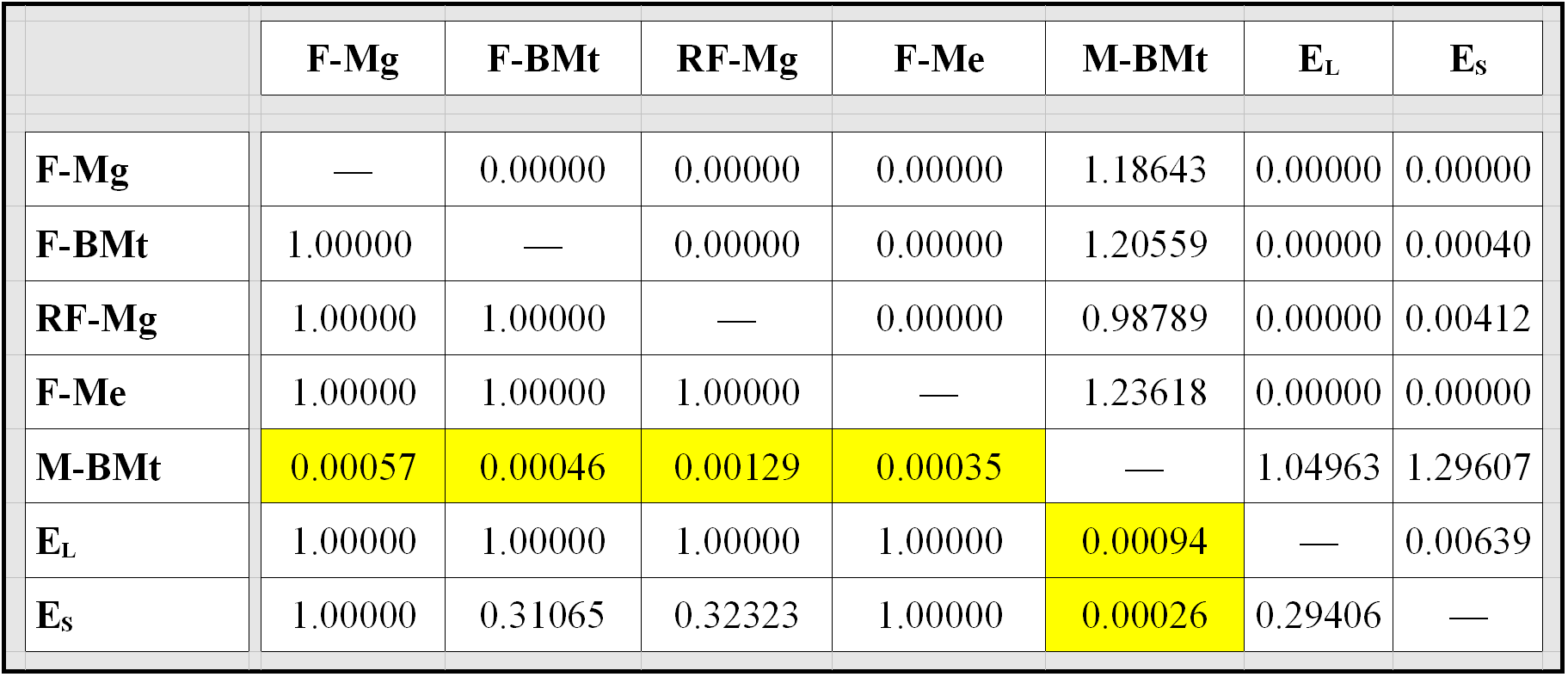

Supplement: Additional file 4: — The results of disparity index test (I D ). The test was performed in all pairwise comparisons in MEGA. Test values are above diagonal, statistical support (p values) are under the diagonal The P-values smaller than 0.05 (yellow marked) indicate significant rate heterogeneity. [file 1471-2156-15-28-S4.png]
